# Limited Specificity of Serologic Tests for SARS-CoV-2 Antibody Detection, Benin

**DOI:** 10.3201/eid2701.203281

**Published:** 2021-01

**Authors:** Anges Yadouleton, Anna-Lena Sander, Andres Moreira-Soto, Carine Tchibozo, Gildas Hounkanrin, Yvette Badou, Carlo Fischer, Nina Krause, Petas Akogbeto, Edmilson F. de Oliveira Filho, Anges Dossou, Sebastian Brünink, Melchior A. Joël Aïssi, Mamoudou Harouna Djingarey, Benjamin Hounkpatin, Michael Nagel, Jan Felix Drexler

**Affiliations:** Université Nationale des Sciences, Technologies, Ingénierie et Mathématiques, Cotonou, Benin (A. Yadouleton);; Laboratoire des Fièvres Hémorragiques Virales du Benin, Cotonou (A. Yadouleton, C. Tchibozo, G. Hounkanrin, Y. Badou);; Charité-Universitätsmedizin Berlin, corporate member of Freie Universität Berlin, Humboldt-Universität zu Berlin, and Berlin Institute of Health, Berlin, Germany (A.-L. Sander, A. Moreira-Soto, C. Fischer, N. Krause, E.F. de Oliveira Filho, S. Brünink, J.F. Drexler);; Ministry of Health, Cotonou (P. Akogbeto, A. Dossou, B. Hounkpatin);; Conseil National de Lutte contre le VIH-Sida, la Tuberculose, le Paludisme, les IST et les Epidémies, Cotonou (M.A. Joël Aïssi);; World Health Organization Regional Office for Africa, Health Emergencies Programme, Brazzaville, Congo (M.H. Djingarey);; Deutsche Gesellschaft für Internationale Zusammenarbeit, Bonn, Germany (M. Nagel);; German Centre for Infection Research, associated partner Charité-Universitätsmedizin, Berlin, Germany (J.F. Drexler)

**Keywords:** respiratory infections, severe acute respiratory syndrome coronavirus 2, SARS-CoV-2, SARS, COVID-19, coronavirus disease, zoonoses, viruses, coronavirus, malaria, parasites, Benin, West Africa

## Abstract

We used commercially available ELISAs to test 68 samples from coronavirus disease cases and prepandemic controls from Benin. We noted <25% false-positive results among controls, likely due to unspecific immune responses elicited by acute malaria. Serologic tests must be carefully evaluated to assess coronavirus disease spread and immunity in tropical regions.

Since its emergence in China late 2019, coronavirus disease (COVID-19) had caused >41 million cases and >1.1 million deaths globally by October 2020, according to the World Health Organization (https://www.who.int/publications/m/item/weekly-operational-update---30-october-2020). Diagnosis of the causative pathogen, severe acute respiratory syndrome coronavirus 2 (SARS-CoV-2), is based on reverse transcription-PCR (RT-PCR) to detect viral nucleic acid or serologic assays to detect SARS-CoV-2 antigens in early stages of disease (*1*,*2*). In later stages of disease, antibody-based serologic testing can complement diagnosis of SARS-CoV-2 infection. In addition, antibody-based serologic testing is a valuable epidemiologic tool to assess COVID-19 spread and potential immunity to SARS-CoV-2. Serologic studies in Europe and Asia indicate high sensitivity and specificity of widely used SARS-CoV-2 antibody ELISAs (*3*,*4*). However, many serologic tests have not been validated in resource-limited settings (*5*). We conducted a SARS-CoV-2 serologic assessment in Benin by using samples from patients with RT-PCR–confirmed SARS-CoV-2 infection and controls sampled before the first SARS-CoV-2 detection in March 2020.

## The Study

We obtained convalescent serum samples from 8 patients in Benin with RT-PCR–confirmed COVID-19 during March–April 2020. The average sampling time was 8 (range 1–10) days after RT-PCR confirmation of SARS-CoV-2 infection (Table 1). We also included 60 serum samples from patients with acute febrile illness tested as part of hemorrhagic fever surveillance during October–November 2019 as prepandemic controls (Table 2). Sampling was approved by the ethics committee of the Benin Ministry of Health (approval no. 030/MS/DC/SGM/DNSP/CJ/SA/027SGG2020).

**Table 1:** Characteristics of patients with RT-PCR–confirmed SARS-CoV-2 infection from whom serum samples were collected during March–April 2020 in Benin*

Sample ID	Age, y/sex	Sampling month	Location	Travel history	Symptoms	Day serum sample taken after RT-PCR–confirmed SARS-CoV-2 infection
1	36/M	March	Cotonou	France	Fever	8
2	43/M	March	Cotonou	Niger	Fever	1
3	34/F	March	Cotonou	France	Fever	8
4	29/M	March	Cotonou	France	Fever	10
5	44/M	April	Cotonou	Germany	Fever	10
6	39/F	April	Cotonou	France	Fever	9
7	41/F	April	Cotonou	France	Fever	8
8	37/M	April	Cotonou	Germany	Fever	8
*ID, identification; RT-PCR, reverse transcription PCR; SARS-CoV-2, severe acute respiratory syndrome coronavirus 2.						

**Table 2:** Characteristics of prepandemic controls with febrile illnesses of unknown origin from whom samples were collected during October–November 2019 in Benin*

Sample ID	Age, y/sex	Health center	Sampling month	Symptoms
215	28/M	CNHU	October	Fever
311	15/F	CB	October	Fever
312	34/F	CB	October	Fever
313	27/M	CB	October	Fever
314	18/M	CB	October	Fever
315	21/M	CB	October	Fever
316	31/M	CB	October	Fever
317	25/F	CB	October	Fever
318	23/F	CB	October	Fever
319	18/M	CB	October	Fever
320	22/F	CB	October	Fever
321	19/F	CB	October	Fever
322	23/M	CB	October	Fever
323	21/M	CB	October	Fever
324	34/F	CB	October	Fever
325	47/M	CB	October	Fever
326	29/M	CB	October	Fever
327	42/F	CB	October	Fever
328	21/M	CB	October	Fever
329	12/M	CB	October	Fever
330	19/F	CB	October	Fever
331	46/M	CB	October	Fever
332	44/F	CB	October	Fever
333	59/M	CB	October	Fever
334	37/M	CB	October	Fever
335	65/M	CB	October	Fever
336	39/F	CB	October	Fever
337	56/M	CB	October	Fever
338	19/M	CB	October	Fever
339	29/M	CB	October	Fever
201	42/M	CB	November	Fever
202	23/M	CB	November	Fever
203	29/M	CB	November	Fever
204	18/M	CB	November	Fever
205	30/F	AHC	November	Fever
206	26/F	AHC	November	Fever
207	19/M	AHC	November	Fever
208	25/F	AHC	November	Fever
209	34/F	AHC	November	Fever
210	61/F	AHC	November	Fever
211	18/F	AHC	November	Fever
212	32/M	AHC	November	Fever
213	63/F	AHC	November	Fever
214	40/M	AHC	November	Fever
216	50/F	CNHU	November	Fever
217	38/M	CNHU	November	Fever
218	55/M	CNHU	November	Fever
219	13/F	CNHU	November	Fever
220	12/F	CNHU	November	Fever
221	29/F	CNHU	November	Fever
222	35/M	CNHU	November	Fever
223	22/M	CNHU	November	Fever
224	15/M	CNHU	November	Fever
225	19/M	CNHU	November	Fever
226	33/F	CNHU	November	Fever
227	16/F	CNHU	November	Fever
228	26/M	CNHU	November	Fever
229	31/F	CNHU	November	Fever
230	26/F	CNHU	November	Fever
291	29/F	AHC	November	Fever

*AHC, Akkasato Health Center; CB, Clinique Boni; CNHU, Centre National Hospitalier Universitaire Hubert Koutoukou; ID, identification.

 We tested all 68 serum samples by using commercially available ELISAs from EUROIMMUN (https://www.euroimmun.com) that rely on different antigens and antibody classes: SARS-CoV-2 nucleocapsid (N) antigen (IgG), spike 1 (S1) subunit (IgG and IgA), and Middle East respiratory syndrome coronavirus (MERS-CoV) S1 (IgG). We also used the SCoV-2 Detect IgG ELISA (InBios, https://inbios.com), an IgG-only S1 antigen-based test authorized for emergency use by the US Food and Drug Administration. Serum samples also were tested by using commercially available ELISA kits (Euroimmun) against the Zika virus (ZIKV) nonstructural protein 1 (NS1) antigen (IgG), the Epstein-Barr virus (EBV) nuclear antigen 1 (EBNA1) (IgG), and the EBV viral capsid (CA) antigen (IgM and IgG), as well as real-time PCR tests (TIB MOLBIOL, https://www.tib-molbiol.com) for all human pathogenic *Plasmodium* species, EBV, and cytomegalovirus (CMV). Plaque-reduction neutralization tests (PRNTs) were performed by using similar methods for SARS-CoV-2 and ZIKV as described (*4*,*6*). We used previously described recombinant S-based immunofluorescence assays (*7*) to test for specific antibodies to common cold betacoronavirus human coronavirus (HCoV) OC43 and HCoV-HKU1.

**Figure 1:**
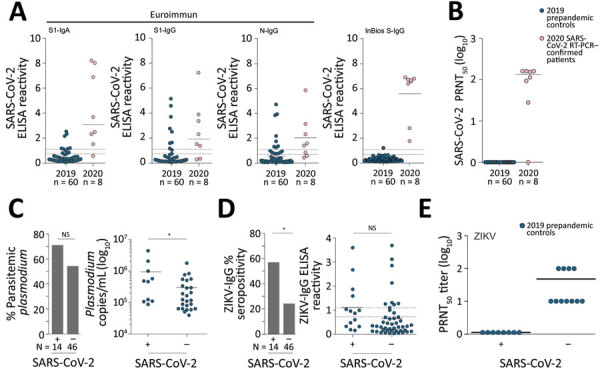
Serologic diagnostics of SARS-CoV-2 and co-existing pathogens in Benin. A) SARS-CoV-2 ELISA reactivity by using different commercially available assays in prepandemic controls from 2019 and SARS-CoV-2 RT-PCR-confirmed patients from 2020. Dashed lines denote the ratio thresholds of >1.1 (positive) and <0.9 (negative); results between these values are considered borderline, as defined by the manufacturers, EUROIMMUN (https://www.euroimmun.com) and InBios (https://inbios.com). Solid line denotes mean ELISA reactivity. B) SARS-CoV-2 PRNT_50_ in prepandemic controls from 2019 and SARS-CoV-2 RT-PCR–confirmed patients from 2020, shown in log_10_ scale for clarity. Solid line denotes mean PRNT log_10_ titer. C) Percentage of prepandemic controls with *Plasmodium* parasitemia who were SARS-CoV-2 ELISA–positive versus those who were SARS-CoV-2 ELISA-negative, shown in log_10_ scale for clarity. Solid line denotes the mean copies/mL. Asterisk denotes p<0.05. D) ZIKV ELISA IgG ELISA percent seropositivity and ZIKV ELISA reactivity within SARS-CoV-2–positive and SARS-CoV-2–negative prepandemic controls. Continuous line denotes the mean ELISA reactivity. Asterisk denotes p<0.05. E) ZIKV PRNT_50_ results. Continuous line denotes the mean PRNT_50_ log_10_ reactivity. NS, not statistically significant; PRNT_50_, 50% plaque reduction neutralization test; SARS-CoV-2, severe acute respiratory syndrome coronavirus 2; ZIKV, Zika virus.

Among the 8 patients with RT-PCR–confirmed SARS-CoV-2 infection, seroconversion ranged from 62.5%–100% (95% CI 30.8%–100.0%), depending on the ELISA used (Figure 1, panel A), suggesting differential sensitivity of ELISAs on the basis of immunoglobulin detected and the commercial kit used. Indeed, early after infection, IgA-based tests had a higher sensitivity than most IgG-based SARS-CoV-2 ELISAs; only the InBios IgG-based kit was positive for all RT-PCR–confirmed patients (Figure 1, panel A). A total of 87.5% (7/8) of ELISA results were confirmed by a highly specific SARS-CoV-2 PRNT (Figure 1, panel B). 

When summarizing all antibody classes, antigens, and kits among the 60 prepandemic controls, we observed 25.0% (15/60; 95% CI 15.7%–37.3%) positive or borderline ELISA results (*8*). Different from RT-PCR–confirmed cases, ELISA reactivity in those samples contrasted with the complete lack of SARS-CoV-2–specific neutralizing antibodies, suggesting unspecific ELISA reactivity (Figure 1, panel B). 

**Figure 2:**
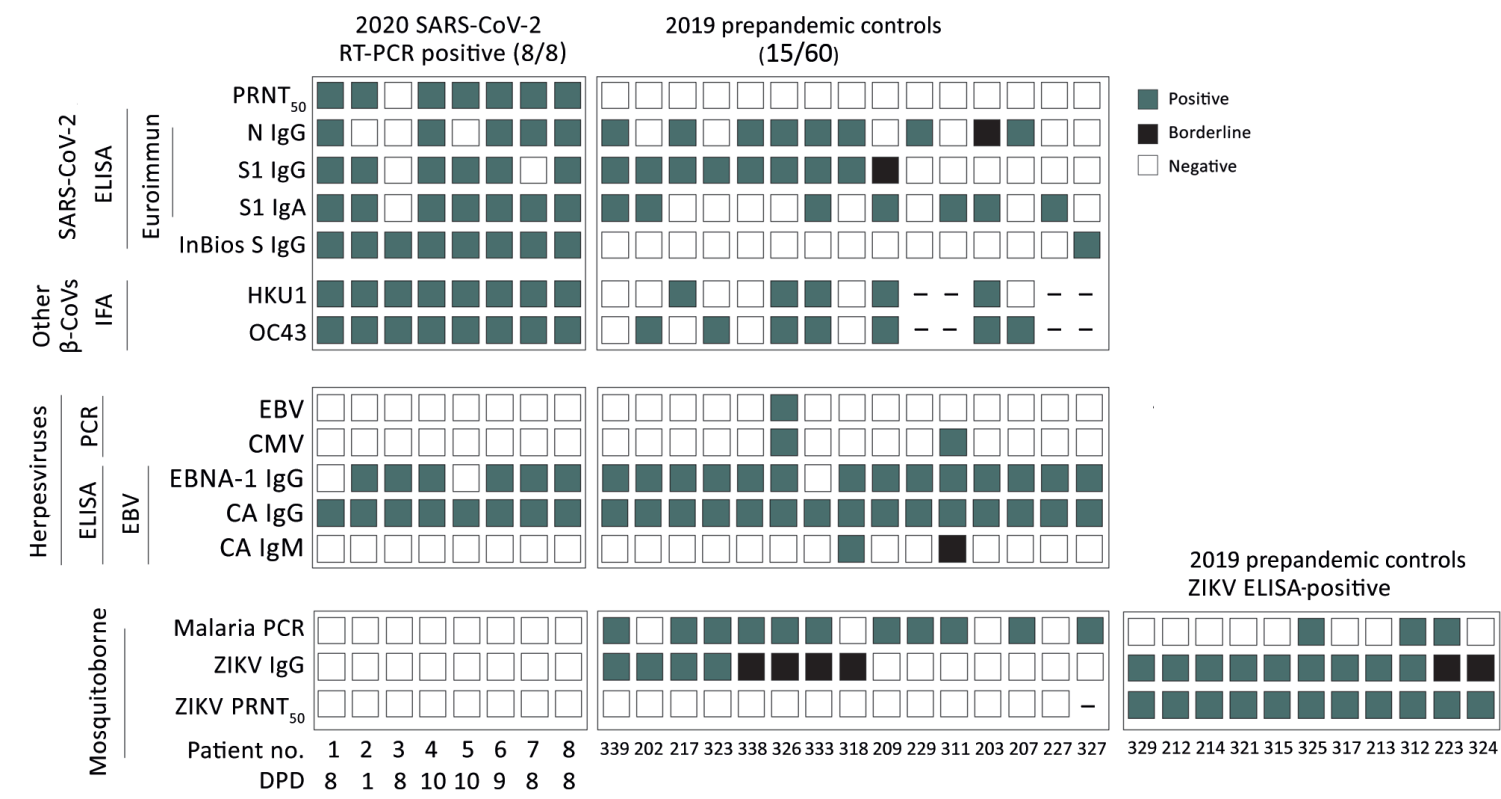
Molecular and serologic test results for betacoronaviruses and co-existing pathogens in Benin. Individual results are shown for reactivity of different commercially available SARS-CoV-2 ELISAs, SARS-CoV-2 PRNT, and IFA reactivity to common cold human coronaviruses OC43 and HKU1 in prepandemic controls from 2019 and SARS-CoV-2 RT-PCR confirmed patients from 2020; EBV PCR, CMV PCR, and 3 EBV ELISAs (EBV-CA IgM, EBV-CA IgG, and EBV-EBNA IgG) from the same groups; and ZIKV-IgG ELISA, ZIKV-PRNT, and malaria PCR from the same groups. Gray squares denote positive results; black squares denote inconclusive results; and white squares denote negative results. Dash (–) denotes samples in which the assay was not performed due to low sample volume. β-CoVs, betacoronaviruses; CA, viral capsid; CMV, cytomegalovirus; DPD, days the serum sample was taken after positive RT-PCR SARS-CoV-2 diagnosis; EBNA, nuclear antigen 1; EBV, Epstein-Barr virus; IFA, immunofluorescence; PRNT_50_, 50% plaque reduction neutralization test; RT-PCR, reverse transcription PCR; SARS-CoV-2, severe acute respiratory syndrome coronavirus 2; ZIKV, Zika virus.

Unspecific SARS-CoV-2 ELISA reactivity might be consistent with, but not limited to, 3 scenarios. First, antibodies elicited by common infections with endemic human coronaviruses might cross-react with SARS-CoV-2 antigens (*1*). However, a Fisher exact test showed no statistically significant difference in the frequency of antibody reactivity with common cold coronavirus antigens between SARS-CoV-2 ELISA-positive serum samples compared with SARS-CoV-2 ELISA-negative samples. In detail, reactivity with HCoV-OC43 was 63.6% in SARS-CoV-2 ELISA-positive samples and 70.4% in SARS-CoV-2 ELISA-negative samples (p = 0.7); reactivity with HCoV-HKU-1 was 45.7% in SARS-CoV-2 ELISA-positive samples and 74.0% in SARS-CoV-2 ELISA-negative samples (p = 0.1) (Appendix Figure 1, panel A). Similarly, a Student *t*-test revealed no statistically significant difference in the magnitude of antibody titers against common cold coronaviruses between SARS-CoV-2 ELISA-positive or ELISA-negative samples (p = 0.09 for HCoV-OC43 and p = 0.8 for HCoV-HKU1) (Appendix Figure 1, panel B). Of note, no serum reacted with MERS-CoV antigens, suggesting that unspecific reactivity might not apply to all coronavirus antigens and tests (Appendix Figure 2). Second, polyclonal B-cell activation can occur in infections with or reactivations of herpesviruses, such as CMV and EBV, and elicit false-positive results in serologic tests (*9*). However, only 2 patients had a positive CMV PCR and only 1 patient had a positive EBV PCR (Figure 2). In addition, persons with SARS-CoV-2 ELISA-positive versus ELISA-negative results did not differ in their past exposure to EBV, according to detailed serologic analyses (Figure 2; Appendix Figure 3). Finally, polyclonal B cell activation also can be caused by acute malaria, which is widespread in Africa (*10*). More (71.4%) persons with SARS-CoV-2–positive ELISAs than those with negative ELISAs (54.3%) were positive for *Plasmodium* in a highly sensitive PCR test, but the difference was not statistically significant by Fisher exact test (p = 0.35; Figure 1, panel C). However, parasite loads were statistically significantly higher among SARS-CoV-2 ELISA-positive than ELISA-negative persons by Student *t*-test (p = 0.035; Figure 1, panel C). In malaria, higher parasite loads are detected at early stages of infection and decrease over time, suggesting a higher proportion of acute malaria in SARS-CoV-2 ELISA–positive patients compared with likely subacute or chronic malaria in SARS-CoV-2 ELISA–negative patients (*11*). Thus, acute malaria is the most plausible explanation for unspecific SARS-CoV-2 ELISA reactivity in prepandemic controls. To assess the breadth of unspecific reactivity, we tested the serum samples from prepandemic controls by using a ZIKV IgG ELISA, for which unspecific reactivity has been reported in cases of acute malaria (*10*). We found that 57.1% of samples that elicited potentially unspecific SARS-CoV-2 ELISA results also showed ZIKV ELISA–positive results, whereas only 23.9% of samples that were SARS-CoV-2 ELISA–negative were ZIKV ELISA–positive. This difference was statistically significant by Fisher exact test (p = 0.019) (Figure 1, panel D; Appendix Figure 4). From the prepandemic controls that were SARS-CoV-2 ELISA positive, no ZIKV ELISA–positive serum samples showed ZIKV-specific neutralizing antibodies, suggesting unspecific reactivity of those samples in the ZIKV ELISA, similar to the discrepant results of SARS-CoV-2 ELISA and PRNT observed in those serum samples (Figure 1, panel E; Figure 2). 

## Conclusion

We assessed SARS-CoV-2 antibody-based serologic diagnostics in Benin and noted unspecific reactivity in up to 25% of febrile patients, possibly due to acute malaria. Limitations of our study include the small sample size and limited patient metadata. Testing of serum samples for CMV and EBV by PCR might not have been sensitive due to lack of cell-associated viral nucleic acid; therefore, we cannot exclude potential herpesvirus reactivation affecting serologic testing. Nevertheless, our analyses point to acute malaria as the likely cause of the unspecific serologic reactivity, although we cannot exclude other coexisting conditions in the tropics, such as dengue virus, which also can affect testing (*12*).

Unspecific reactivity in serologic tests might affect public health interventions in tropical regions, leading to overestimates of SARS-CoV-2 circulation in regions where malaria is endemic and to misidentification of SARS-CoV-2 hotspots. In addition, due to false-positive SARS-CoV-2 results, target populations for vaccine campaigns might be missed when vaccines become available, and coexistent diseases, such as malaria, might be overlooked, leading to higher mortality rates from endemic diseases (*13*,*14*). The robustness of current and future SARS-CoV-2 serologic tests should be further assessed by multicentric seroepidemiologic studies from different tropical regions (*15*).

Appendix Additional information on the limited specificity of serologic tests for SARS-CoV-2 antibody detection, Benin.
