# Supplementary material for: Limited Specificity of Serologic Tests for SARS-CoV-2 Antibody Detection, Benin
Source: Emerg Infect Dis. 2021 Jan;27(1):233–7. doi: 10.3201/eid2701.203281 (PMC7774555; doi:10.3201/eid2701.203281)
Supplement: Appendix — Additional information on the limited specificity of serologic tests for SARS-CoV-2 antibody detection, Benin. [file 20-3281-Techapp-s1.pdf]

Article DOI: https://doi.org/10.3201/eid2701.203281

Limited Specificity of Serologic Tests for SARS-CoV-2 Antibody Detection, Benin, West Africa

Appendix

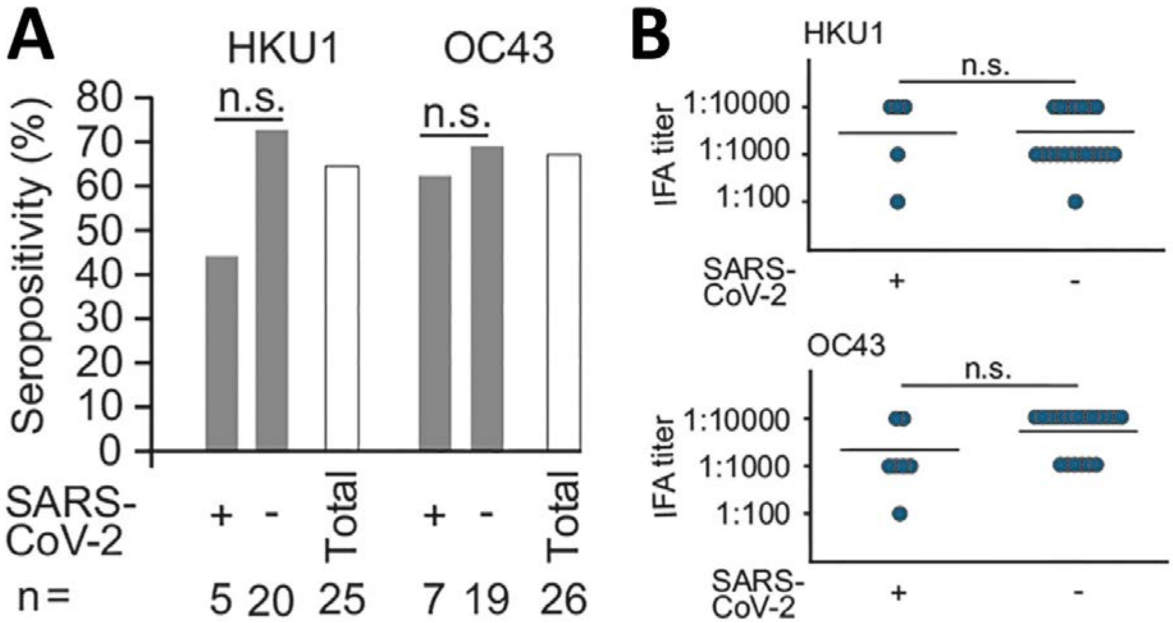

Appendix Figure 1. Serologic diagnostics of common cold betacoronaviruses, Benin, West Africa. A) Common cold betacoronaviruses HKU1 and OC43 seropositivity of SARS-CoV-2 ELISA-positive compared with ELISA-negative serum samples collected from prepandemic controls in 2019. B) Common cold betacoronavirus IFA titers in samples collected from prepandemic controls in 2019 and SARS-CoV-2 RT-PCR–confirmed patients in 2020. Negative samples are not shown for graphic reasons. n.s., not statistically significant, RT-PCR, reverse transcription-PCR; SARS-CoV-2, severe acute respiratory syndrome coronavirus 2.

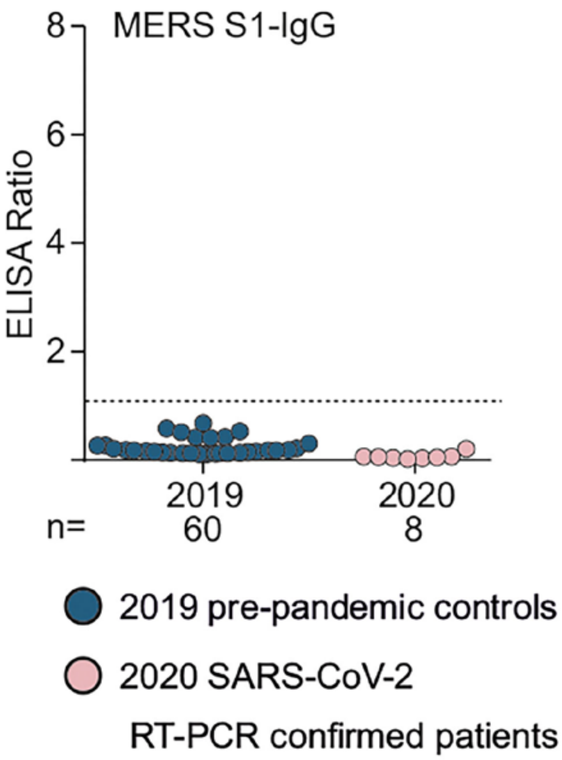

Appendix Figure 2. MERS-CoV ELISA ratio among serum samples collected from prepandemic controls in 2019 and SARS-CoV-2 RT-PCR–confirmed patients in 2020. Dashed line denotes the ratio of the positivity threshold >1.1 as defined by the manufacturer, EUROIMMUN (https://www.euroimmun.com). MERS-CoV, Middle East respiratory syndrome coronavirus; RT-PCR, reverse transcription-PCR; SARS-CoV-2, severe acute respiratory syndrome coronavirus 2.

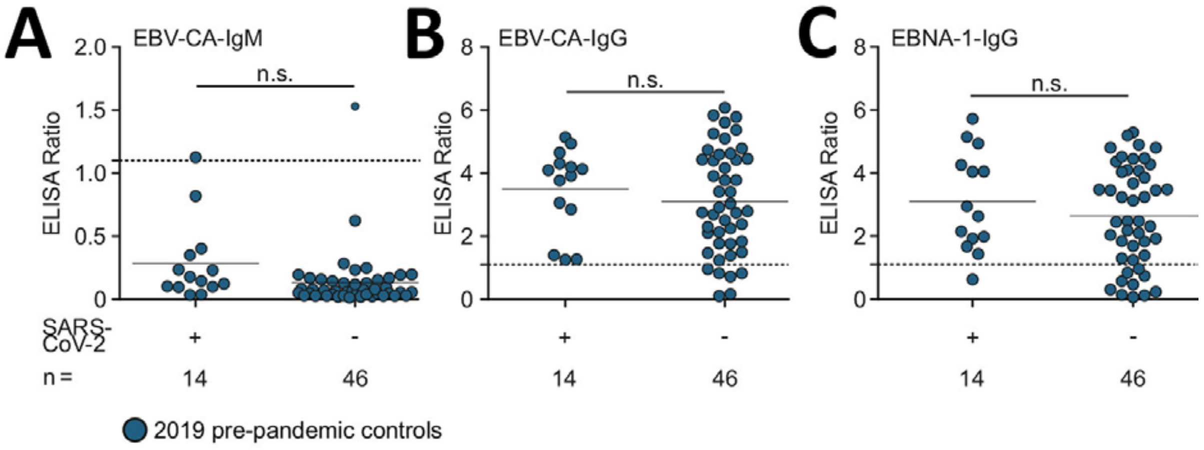

Appendix Figure 3. Molecular and serologic test results for A) EBV-CA IgM, B) EBV-CA IgG, and C) EBV-EBNA1 IgG ELISA ratio among serum samples collected from prepandemic controls in 2019 that were SARS-CoV-2 ELISA-positive versus those that were ELISA-negative, Benin. Dashed lines denote the ratio positivity threshold defined by the manufacturer, EUROIMMUN (https://www.euroimmun.com). Continuous line denotes the mean ELISA reactivity. EBV, Epstein-Barr virus; n.s., not statistically

significant; RT-PCR, reverse transcription-PCR; SARS-CoV-2, severe acute respiratory syndrome coronavirus 2; nuclear antigen 1, EBNA1; viral capsid, CA

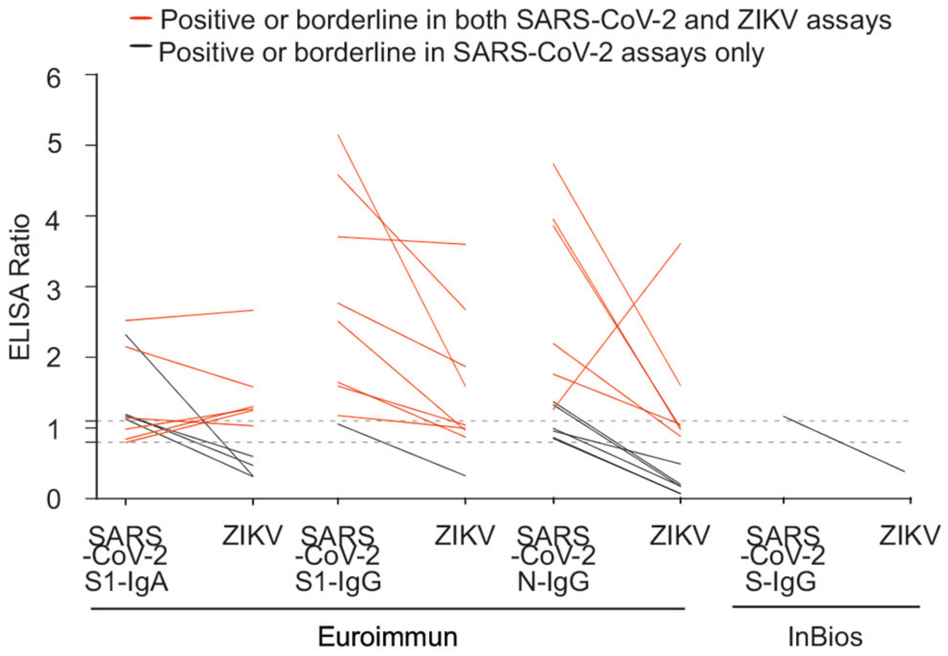

Appendix Figure 4. ELISA ratio comparison between SARS-CoV-2 S1-IgA, S1-IgG, N-IgG ELISA and InBios SCoV-IgG ELISA positive or borderline patients with ZIKV-IgG ELISA. Thin dashed line denotes the ratio positivity threshold of >1.1; thick dashed line denotes the lower threshold limit of <0.9; results between $\geq$0.9 to $\leq$1.1 are considered borderline, as defined by the manufacturers, EUROIMMUN (https://www.euroimmun.com) and InBios (https://inbios.com). N, nucleocapsid; S, spike; SARS-CoV-2, severe acute respiratory syndrome coronavirus 2; ZIKV, Zika virus.
